# Diversity, Transmission, and Cophylogeny of Ledanteviruses (*Rhabdoviridae*: *Ledantevirus*) and Nycteribiid Bat Flies Parasitizing Angolan Soft-Furred Fruit Bats in Bundibugyo District, Uganda

**DOI:** 10.3390/microorganisms8050750

**Published:** 2020-05-17

**Authors:** Andrew J. Bennett, Adrian C. Paskey, Jens H. Kuhn, Kimberly A. Bishop-Lilly, Tony L. Goldberg

**Affiliations:** 1 Department of Pathobiological Sciences, University of Wisconsin-Madison, Madison, WI 53706, USA; abennett3@wisc.edu; 2 Uniformed Services University of the Health Sciences, Bethesda, MD 20814, USA; adrian.paskey@usuhs.edu; 3 Leidos, Reston, VA 20190, USA; 4 Genomics and Bioinformatics Department, Biological Defense Research Directorate, Naval Medical Research Center—Frederick, Fort Detrick, MD 21702, USA; kimberly.a.bishop-lilly.civ@mail.mil; 5 Integrated Research Facility at Fort Detrick, National Institute of Allergy and Infectious Diseases, National Institutes of Health, Fort Detrick, Frederick, MD 21702, USA; kuhnjens@niaid.nih.gov; 6 Global Health Institute, University of Wisconsin-Madison, Madison, WI 53706, USA

**Keywords:** rhabdovirus, ledantevirus, ectoparasite, cophylogenetics, metagenomics

## Abstract

Obligate hematophagous ectoparasitic flies of the superfamily Hippoboscoidea are distributed worldwide, but their role as vectors and reservoirs of viruses remains understudied. We examined hippoboscoid bat flies (family Nycteribiidae) parasitizing Angolan soft-furred fruit bats (*Lissonycteris angolensis ruwenzorii*) from Bundibugyo District, Uganda. Using metagenomic methods, we detected 21 variants of the rhabdovirid genus *Ledantevirus*, which contains medically important “bat-associated” viruses. These 21 viruses, representing at least two divergent viral lineages, infected 26 bat flies from 8 bats in a single roost. Cophylogenetic analyses of viruses and bat flies resulted in strong evidence of virus-host codivergence, indicating vertical transmission of bat fly ledanteviruses. Examination of oral swabs from bats revealed ledantevirus RNA in the saliva of 1 out of 11 bats, with no evidence of insect genetic material in the mouth of this bat. These data demonstrate that bat flies can harbor diverse ledanteviruses even in a single roost and that the predominant mode of transmission is likely vertical (among bat flies), but that bats can become infected and shed viruses orally. In conclusion, bat flies may serve as ectoparasitic reservoirs of “bat-associated” viruses that only transiently or sporadically infect bats.

## 1. Introduction

Flies of the superfamily Hippoboscoidea are globally distributed obligate dipteran ectoparasites of vertebrates. The hippoboscoid family Nycteribiidae contains eyeless, wingless parasites of bats with relatively high host specificity [1]. In recent years, diverse viruses have been discovered in nycteribiid bat flies. For example, Mahlapitsi virus (MAHLV), a fusogenic orthoreovirus (*Reoviridae*: *Spinareovirinae*), was detected in the nycteribiid *Eucampsipoda africana* Theodor, 1955 parasitizing Egyptian rousettes (*Rousettus aegyptiacus* E. Geoffroy, 1810) in Limpopo Province, South Africa [2]. Two novel orthobunyaviruses (*Bunyavirales*: *Peribunyaviridae*) were detected in *Eucampsipoda africana* parasitizing Egyptian rousettes in South Africa (Wolkberg virus [WBV]) [3] and *Eucampsipoda sundaica* Theodor, 1955 parasitizing Leschenault’s rousettes (*Rousettus leschenaultii* Desmarest, 1820) in Yúnnán Province, China (Kaeng Khoi virus; KKV) [4,5]. We previously described Kanyawara virus (KYAV), a member of genus *Ledantevirus* (*Mononegavirales*: *Rhabdoviridae*) in unspecified dipseliopod nycteribiid bat flies parasitizing unspecified collared fruit bats (*Myonycteris* sp.) in Kabarole District, Uganda [6].

Ledanteviruses are subdivided into 3 subgroups (A–C) each containing closely related members with similar host associations and ecologies [7]. KYAV and Wǔhàn louse fly virus 5 (WhLFV-5) [8], discovered in an unidentified hippoboscid fly in Húběi Province, China, joined the “bat-associated” ledantevirus subgroup C (Mount Elgon bat virus (MEBV), Fikirini bat virus (FKRV), Oita virus (OITAV), Kolente virus (KOLEV), Kumasi virus (KRV)) [7], providing a phylogenetic link between hippoboscid fly and “bat-associated” ledanteviruses.

Bats (Chiroptera) host a large number of viruses. This fact has been attributed to their unique immune systems, behavior, and ecology [9,10]. However, the mechanisms by which many viruses are subclinically maintained in bats remain unclear. For example, some “bat-associated” viruses are found sporadically in bats or have been associated with bats only circumstantially [11]. Evidence is growing that bat flies may maintain these viruses; not necessarily as vectors, but rather as primary reservoirs, with only sporadic or transient infection of bats through bat fly bites [6,12,13]. Bat flies also bite humans, providing an alternative explanation for how certain “bat-associated” viruses may cause occasional zoonotic infections [1,6].

In Summer 2017, we sampled Angolan soft-furred fruit bats (*Lissonycteris angolensis ruwenzorii* Bocage, 1898) and their bat flies in Bundibugyo District, Western Region, Uganda, west of the Ruwenzori Mountains on the border with the Democratic Republic of the Congo. Bats were sampled from a single roost. Broad-spectrum metagenomic methods were used to investigate the bats and their ectoparasites for virus infections, and near-complete bat fly mitochondrial genome DNA (mtDNA) sequences were used to examine relationships among bat flies. We were thus able to characterize the diversity of bat fly viruses and the congruence of host and viral phylogenies to test hypotheses about horizontal and vertical viral transmission. Our findings expand the known diversity of bat fly viruses and offer new insights into the role of bat flies as both reservoirs and vectors of “bat-associated” viruses.

## 2. Materials and Methods

### 2.1. Collection of Samples

The site of sample collection (0°50’18.5”N and 30°10’22.1”E), was a stone and cement culvert that serves as drainage for an unnamed Ruwenzori Mountain valley stream bridged by the Itojo-Sempaya Old Road linking Bundibugyo (24 km southwest) in Bundibugyo District with Fort Portal in Kabarole District, Uganda. This site is a continuously occupied, multi-species roost containing several hundred Angolan soft-furred fruit bats (*Lissonycteris angolensis ruwenzorii* Bocage, 1898) and a population of Sundevall’s leaf-nosed bats (*Hipposideros caffer* Sundevall, 1846). Bats were collected as they exited the roost at dusk by mist-net (Avinet, Portland, ME, USA) and were held in cloth bags until sampling.

Oral samples were collected from each bat using sterile rayon-polyester-tipped collection swabs and were preserved in TRI Reagent (Zymo Research, Irvine, CA, USA). Bats were visually screened for ectoparasites. 26 bat flies were removed from 8 infested bats using sterile forceps and immediately placed in sterile tubes (one tube per bat), each containing 500 μL of RNAlater nucleic acid stabilization solution (ThermoFisher Scientific, Waltham, MA, USA). Bats were offered sugar/water solution (30% m/v) following sample collection and were released on site. Oral swabs and bat fly samples were frozen at −20 °C within 3 h of sample collection. All samples were exported with permission of the Uganda Wildlife Authority and the Uganda National Council for Science and Technology, shipped in accordance with International Air Transport Association (IATA) regulations, and imported under permit number 2017-07-103 from the Centers for Disease Control and Prevention (Atlanta, GA, USA).

### 2.2. Metagenomics for Virus Identification

Prior to nucleic acid extraction, bat flies were removed from RNAlater and surface-cleaned with 70% ethanol solution to remove excess RNAlater and environmental contaminants [14]. Total RNA was then extracted and libraries were prepared for high throughput sequencing (HTS) as previously described [6]. Briefly, sequencing libraries were prepared from 1 ng of cDNA per sample using the Nextera XT DNA sample kit (Illumina, San Diego, CA, USA) and sequenced using the Illumina MiSeq platform (Illumina, San Diego, CA, USA). HTS reads were quality-trimmed and contiguous sequences (contigs) were de novo assembled using CLC Genomics Workbench version 11.1 (CLC Bio, Aarhus, Denmark) as previously described [6]. Assembled contigs were compared at the nucleotide and deduced protein sequence level to virus-derived sequence in GenBank using BLASTn and BLASTx homology searching algorithms, respectively [15].

RNA was extracted from bat oral swab samples using the Direct-zol RNA MicroPrep kit (Zymo Research, Irvine, CA, USA). Illumina RNA TruSeq libraries were prepared, and sequenced using Illumina NextSeq 500 v2 chemistry (Illumina, San Diego, CA, USA). Viral contigs were identified using the VirusSeeker discovery pipeline [16] and confirmed by analysis of metaSPAdes v3.11.1 de novo assembled contigs [17]. Classification of contigs was performed by DIAMOND using the blastX algorithm against RefSeq viral protein sequences [18].

### 2.3. Ledantevirus Phylogenetics

We detected fragments of the large protein (L) gene of a ledantevirus in one bat oral swab. We chose a conserved but phylogenetically informative 270-base region within this sequence for phylogenetic reconstruction because we were unable to resolve the full virus genome from this sample due to low concentrations of extracted nucleic acids. We aligned sequences using the Prank algorithm [19] implemented in TranslatorX [20]. We then constructed a maximum likelihood phylogenetic tree of bat fly ledanteviruses and the viral fragment from the bat oral swab, including representatives of the ledanteviruses, and 3 vesiculoviruses (which are related rhabdovirids) as an outgroup. The phylogeny was inferred using PhyML with Akaike information criterion smart model selection “general-time reversible model with incorporation of rate of variation across site and proportion of invariable sites” (GTR+ Γ+I) [21].

### 2.4. Bat Fly Mitogenome Sequencing

To estimate relationships among bat flies, and for use in co-phylogenetic analyses, we constructed a reference mitochondrial genome (mitogenome) from one nycteribiid bat fly and annotated the genome using the MITOS web server [22]. We confirmed open reading frames for canonical genes using BLASTn, and mapped sequencing reads and contigs from the remaining bat flies to this reference mitogenome with high stringency (length fraction 0.9, similarity fraction 0.95). We then extracted sequences of seven mitochondrial genes (NADH dehydrogenase subunit 1–3 (*NAD1–3*), cytochrome C oxidase subunit 1–3 (*COX1–3*), and cytochrome B (*CYTB*) from each individual bat fly mitogenome (GenBank Accession numbers MT293653–MT293772 and MT278266–MT278285) and concatenated the sequences to create a 6519-bp sequence alignment. We then used this alignment to infer a bat fly phylogeny with best-fit model of molecular evolution (HKY85+I) estimated by PhyML smart model selection “Bayesian Information Criterion” (BIC).

### 2.5. Identifying Patterns of Selection Across Viral Genomes

We aligned coding-complete genomes of 21 ledanteviruses (GenBank Accession numbers MT325641–MT325661) from the Bundibugyo District bat fly population using the Prank algorithm implemented in TranslatorX. We then concatenated coding sequences, removing intergenic and terminal untranslated regions, and estimated non-synonymous (dN) and synonymous (dS) substitution rates across the genome alignment using SNAP (Synonymous Non-synonymous Analysis Program) v2.1.1 [23]. We calculated the dN/dS ratio across the genome using a sliding window (100-aa window, 20-aa step) as previously described [24]. We then aligned the 21 ledantevirus nucleotide and amino acid sequences with the nearest outgroup, the ledantevirus Mount Elgon bat virus (MEBV) (RefSeq #NC_034545.1). We calculated overall mean distance and standard error (using 1000 bootstraps) and pairwise p-distances among viruses using MEGA7 [25].

### 2.6. Cophylogenetic Analyses

We assessed congruence between virus and bat fly phylogenies as evidence for vertical transmission of viruses in the Bundibugyo District roost. Because mtDNA is maternally inherited [26], correspondence of virus and arthropod phylogenies would support vertical transmission, whereas lack of correspondence would support other modes of transmission. We first generated codon-based alignments for concatenated coding sequences of 20 viral genomes using the Prank algorithm implemented in TranslatorX and inferred the mtDNA phylogeny as described above. We inferred the virus phylogeny using the best-fit model of molecular evolution (Hasegawa, Kishino, and Yano [HKY85]+G+I) estimated by PhyML smart model selection (BIC). We also inferred phylogenies from individual viral genes to assess differences between this approach and that obtained from concatenated coding sequences.

We generated a tanglegram of the host and virus phylogenies using TreeMap 3.0b, and tested the global and local significance of cophylogenetic association by comparing the observed correlation between host and virus trees and subtrees to correlations derived from a set of randomized subtrees [27]. We also examined the degree of co-divergence between viruses and bat fly mtDNA using Procrustes application to cophylogenetic analysis (PACo) [28] implemented in R [29]. PACo tests for dependence of a virus phylogeny on that of a host by superimposing principal coordinates generated from the phylogenetic distance matrices of virus and host. We assessed the significance of the observed sum of the squared residuals (m^2^) by comparing the observed values to values obtained from 10,000 randomized virus-host associations.

### 2.7. Episodic Diversifying Selection on Virus Diversity

Based on the results of cophylogenetic analyses, we examined whether episodic diversifying selection might have contributed to the diversification of viruses. We tested the phylogeny of aligned concatenated viral coding sequences for evidence of episodic diversifying selection using an improved branch-site model (adaptive branch-site random effects likelihood; aBSREL) implemented using the datamonkey webserver [30]. We assessed statistical significance of the 35 branches tested using likelihood ratio tests at an alpha threshold of 0.05 with correction for multiple testing.

## 3. Results

### 3.1. Bat Flies and Bat Oral Swabs

We collected oral swabs from 11 bats and harvested their associated ectoparasites. Based on 2 nights of double-observer roost exit counts (preceding the sampling period), we estimate this sample to represent 5.5% of the approximately 200 Angolan soft-furred fruit bats in the roost at the time. Eight of the 11 bats were infested with bat flies, with an average intensity of 3.25 flies per bat (range 2‒5 flies per bat). Nycteribiid bat flies were the only ectoparasites found.

**Figure 1:**
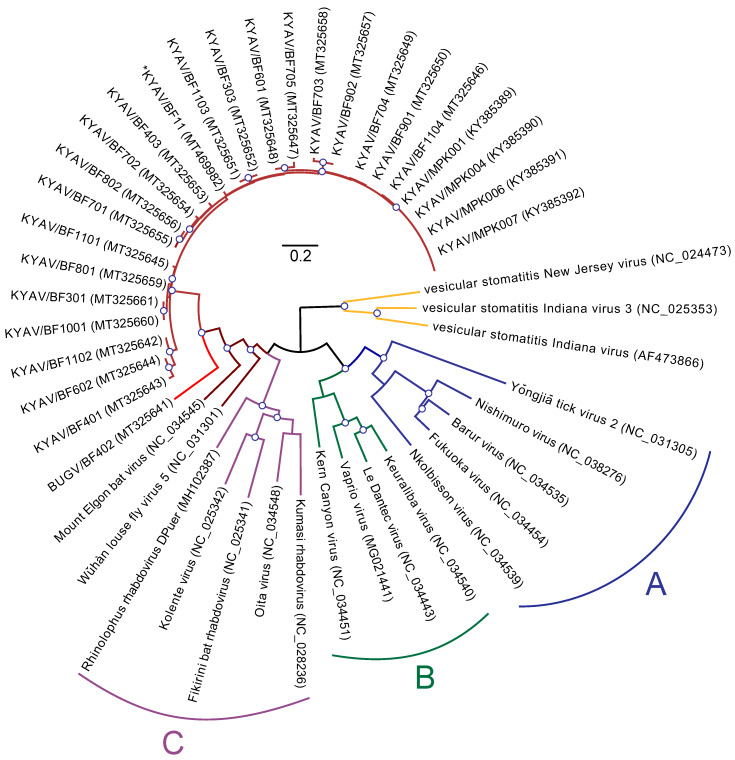
Maximum likelihood tree of the ledantevirus large protein (*L*) gene. Maximum likelihood phylogenetic tree of genus *Ledantevirus* (*Mononegavirales*: *Rhabdoviridae*) with 3 members of the neighboring *Vesiculovirus* genus as an outgroup (yellow). The tree was constructed from a conserved 270-nt sequence of the RNA-directed RNA polymerase domain of the large protein (L), with molecular evolution model selection (GTR+Γ+I) by PhyML smart model selection (Akaike information criterion). Canonical ledantevirus subgroups are highlighted in blue (Subgroup **A**), green (Subgroup **B**), and purple (Subgroup **C**), although Mount Elgon Bat virus, historically of Subgroup C, is included in a monophyletic (red) clade with the hippoboscoid fly ledanteviruses (Kanyawara virus, Bughendera virus, and Wǔhàn louse fly virus 5). Asterisk indicates sequence from bat oral swab. Circles on nodes indicate >85% confidence based on 1000 bootstrap replicates. Scale bar indicates nucleotide substitutions per site.

Metagenomic sequencing of bat flies revealed the presence of viruses of the rhabdovirid genus *Ledantevirus* in 21 of 26 of the flies (80.7%). Sequencing reads were trimmed for quality (Q30), leaving an average 1.02 × 10^6^ (± 1 × 10^5^) reads per sample. An average of 0.46% (± 0.3%) of trimmed reads mapped to a ledantevirus, giving an average coverage depth of 64.85 (± 11.2) reads. Each infected bat fly was host to a single ledantevirus. Twenty of these viruses were closely related to each other and to KYAV (KYAV, maroon, Figure 1), but one was a divergent outgroup to this clade, which we named Bughendera virus (BUGV, scarlet, Figure 1) for the county in which it the virus was discovered (Table 1). Three bat flies (BF401–403) were collected from the bat host (BF4) of the BUGV-infected fly (BF402) and flies BF401 and BF403 were infected with two of the most divergent KYAV variants (pairwise p-distance = 9.2% ± 0.32). The 20 KYAV isolates and BUGV have the same genome organization as the other subgroup C ledanteviruses (nucleoprotein (N), phosphoprotein (P), matrix protein (M), glycoprotein (G), large protein (L)) [6].

Oral swabs of 11 bats were also sequenced, and KYAV virus contigs were detected in the saliva of one bat using VirusSeeker. To examine whether these sequences were present as a result of the bat having ingested a bat fly, we screened trimmed sequencing reads from this bat against an insect ribosomal RNA database [31]. All contigs were also queried against the National Center for Biotechnology Information (NCBI) nucleotide databases using BLASTn, but no insect genetic material was detected using either method.

The MITOS web server analysis of assembled contigs predicted a 15,088-bp mitogenome (Supp. 1). The *COX2* and *CYTB* genes were the most similar (by BLASTn) to partial sequences from dipseliopod bat flies from Kibale National Park, Uganda [6] (99.55% and 100%, respectively). Using this mitogenome as a reference scaffold, we assembled the seven mitochondrial genes used in our co-phylogenetic analyses for the remaining 19 bat flies infected with KYAV.

### 3.2. Phylogeny of Ledanteviruses

Ledanteviruses from Bundibugyo bat flies and a bat oral swab form a clade with KYAV from Kibale National Park, Uganda [6] with BUGV as an outgroup to this clade. KYAV isolates and BUGV, in turn, form a clade with MEBV and WhLFV-5 of ledantevirus subgroup C (brown, Figure 1). By this analysis, BUGV appears to meet International Committee on Taxonomy of Viruses (ICTV) *Rhabdoviridae* Study Group amino acid identity standards for the need to establish a new member species within the genus *Ledantevirus* [32], having an RNA-directed RNA polymerase amino acid sequence divergence of greater than 7% (from KYAV: 28.6%; from MEBV: 37%), and glycoprotein amino acid sequence divergence of >15% (from KYAV: 30.6%; from MEBV: 51.8%). Within the KYAV isolates, average p-distance among viruses was 6.2% ± 0.16%. Topologies inferred from individual viral genes did not differ significantly from those inferred from concatenated genes.

### 3.3. Viral Diversity and Selection

**Figure 2:**
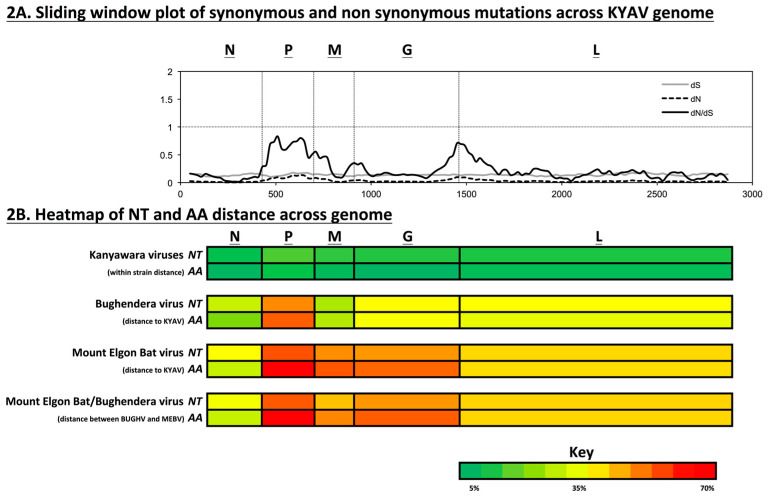
Selection and diversity within KYAV-like viruses and viruses of neighbor taxa. (**A**) Sliding window plot (100-aa window, 20-aa step) of dN/dS across the genome of 20 KYAV-like viruses from Bundibugyo District, Uganda. Genome-wide dN/dS <1, indicating stabilizing selection predominates, but this constraint is relaxed for portions of P, M, and L. (**B**) Average pairwise distances of nucleotide (NT) and amino acid (AA) sequences for each virus gene within the KYAV isolates, between KYAV isolates and BUGV, between KYAV isolates and Mount Elgon bat virus (MEBV), and between BUGV and MEBV are represented by a heat map. For KYAV-like viruses in the population, average pairwise distances varied across genes, and were high for a single population (Avg NT dist: N = 5.12%, P = 9.74%, M = 7.76%, G = 7.34%, L = 7.00%; Avg AA dist: N = 1.30%, P = 6.03%, M = 2.86%, G = 0.77%, L = 4.00%).

**Figure 3:**
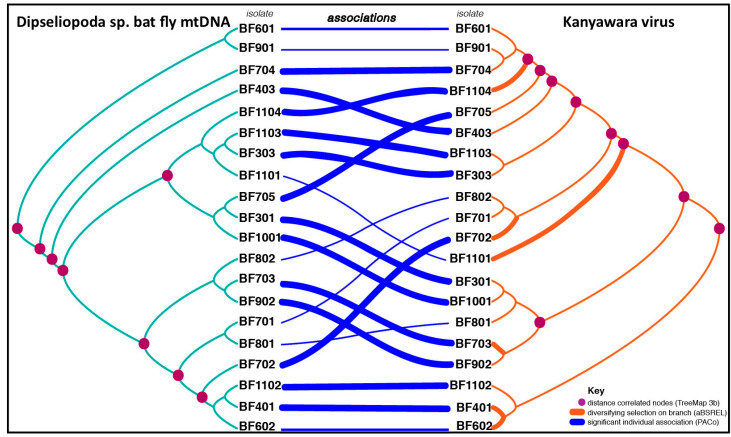
Congruence between bat fly mtDNA and virus phylogeny suggest vertical ledantevirus transmission. Tanglegram generated by TreeMap 3.0b for 7 concatenated bat fly mtDNA genes (NAD1–3, COX1–3, CYTB) (left) and concatenated KYAV-like virus coding sequence (right). Host-virus associations are indicated by crossing blue lines. Individual host-virus links with Procrustes application to cophylogenetic analysis (PACo) procrustean squared residuals below the mean value for all links are believed to contribute significantly to the overall cophylogenetic structure, and are indicated by weighted blue associations lines. TreeMap 3.0b Distance Correlation was performed, and host and virus tree node pairs leading to significantly correlated subtrees (*p* < 0.05) are marked with purple dots. Adaptive branch-site random effects likelihood (aBSREL) detected significant (*p* < 0.05) evidence of episodic diversifying selection on 6 of 37 virus tree branches (indicated by weighted orange lines).

Viral nucleotide and amino acid diversity between hosts (bat flies) were high for the KYAV isolates, and this diversity varied across the genome (Figure 2B). Across the genomes of the KYAV isolates, average dS was consistent (0.14 ± 0.0178 SD), and average dN was lower than the average dS, but showed greater variability (dN = 0.03 ± 0.0255), as expected (Figure 2). The average dN/dS ratio was <1 across the genome (0.23 ± 0.18), indicating a genome-wide pattern of purifying selection. dN/dS varied widely among genes (*N* = 0.089, *P* = 0.617, *M* = 0.284, G = 0.243, *L* = 0.192). However, the aBSREL model found significant evidence of episodic diversifying selection on 6 of 35 branches in the phylogeny of concatenated coding genes of the KYAVs. Diversifying selection was detected on branches leading to KYAV strains BF401, BF703, BF1101, BF702, BF602, BF602, BF1104 (corrected *p*-values: 0.0000, 0.0000, 0.0001, 0.0004, 0.0051, 0.0113, respectively, Figure 3).

### 3.4. Cophylogenetic Analyses

We found significant congruence between host and virus phylogenies by the TreeMap 3.0b distance correlation test (Figure 3). Furthermore, PACo analysis indicated that the KYAV phylogeny is significantly dependent upon the host phylogeny (m^2^ = 0.01326, *p* = 0.00091).

## 4. Discussion

The *Ledantevirus* genus within the family *Rhabdoviridae* was formally constituted by the ICTV in 2017 [32]. The genus was named after its earliest-identified member, Le Dantec virus (LDV), which in 1965 was discovered in the blood of a febrile patient of Le Dantec University Hospital in Senegal [33]. In 1969, LDV was found in a Welsh dockworker who reported receiving a painful bite from an unidentified arthropod while working in the hold of a ship that had traveled from Western Africa. Subsequently, he became ill with fever and neurologic disease [34]. Although the natural host of LDV is unknown, its immediate relatives are strongly associated with bats, rodents, and hematophagous arthropods that may associate with both [7].

Ledanteviruses are nested within the unofficial “dimarhabdovirus supergroup,” which is largely composed of viruses that replicate in both vertebrate and invertebrate cells [35]. Researchers have speculated that ledanteviruses may be vector-borne [7]. In support of this idea, three ledanteviruses have been detected in both vertebrate and invertebrate hosts: Barur virus (BARV) was found in rodents/ticks [36], Kolente virus (KOLEV) was found in bats/ticks [37], and Fukuoka virus (FUKV) was found in mosquitoes/cattle [38,39]. Furthermore, Yǒngjiā tick virus 2 (YTV-2), WhLFV-5 (unidentified hippoboscid fly), and Nkolbisson virus (NKOV) (mosquito) have all been detected in hematophagous arthropods [8,40,41].

KYAV was discovered infecting nycteribiid bat flies infesting collared fruit bats (*Myonycteris* sp.) in Kibale National Park, Uganda [6]. MEBV, the closest relative to KYAV, was isolated from the salivary gland of an eloquent horseshoe bat (*Rhinolophus eloquens* K. Andersen, 1905) in Kenya [42]. MEBV replicates to high titers in experimentally infected *Aedes aegypti* mosquitoes [42], but has not, to our knowledge, been found in an insect in nature. Together, these findings support the notion that ledanteviruses within subgroup C can infect both vertebrates and invertebrates.

Here, we report an extraordinary frequency of infection and diversity of KYAV variants, and a novel ledantevirus, BUGV, in a single population of bat flies from Bundibugyo District, Uganda. This diversity is particularly surprising because the sampled bats were all of the same species, captured at the same time, and all bat flies were of the same genus (*Dipseliopoda*). Eight of 11 sampled bats were infested with bat flies, and 21 of 26 of bat flies were each host to a single ledantevirus (Table 1). Bat flies collected from the same bat did not host more closely related KYAV isolates than would be expected by chance (data not shown). In fact, the sole bat fly infected with the novel BUGV (BF402) was itself most closely related to bat fly BF701 but was found parasitizing a single bat alongside two other bat flies (BF401, BF403) hosting two of the most distantly related KYAV isolates identified.

Virus genome fragments were detected in the saliva of a bat-fly-infested bat, yet we found no evidence of insect consumption, as evidenced by the absence of insect DNA in the oral swab from that bat. This finding reinforces the notion that although subgroup C ledanteviruses primarily infect insects, they occasionally infect the vertebrates with which those insects are associated. If so, bat fly ledanteviruses are unlikely to be maintained in nature through transmission among vertebrate hosts, such as bats. This natural history is not unlike that documented for other viruses, such as La Crosse virus (*Bunyavirales*: *Peribunyaviridae*), a mosquito-borne virus that relies on trans-ovarial vertical transmission and horizontal transmission via amplifying vertebrate hosts [43]. To assess vertical transmission directly, we examined correspondence between fly and virus phylogenies. The significant congruence between virus and the mitochondrial phylogenies supports our hypothesis that KYAV isolates may be vertically transmitted from maternal to offspring flies. Thus, we conclude that bat fly ledanteviruses are not “traditional” obligate arboviruses, but rather are maintained in nature through vertical transmission in their insect reservoirs but with occasional vectored transmission to mammals (bats and, occasionally, humans).

Arboviruses are highly constrained by the physiological demands of replication in vertebrate and invertebrate hosts [44,45]. For example, in a 2004 study, maximum genetic divergence was just 1.1% for 48 isolates of the vector-borne vesicular stomatitis New Jersey virus (VSNJV; *Rhabdoviridae*: *Vesiculovirus*) collected across 1651 km [46]. In contrast, the genetic diversity of Pararge aegeria rhabdovirus, a vertically transmitted unclassified virus isolated from butterflies across the United Kingdom over a 2 month span, reached 2.2% [47]. Bi-parental vertical transmission of this virus would explain this high diversity and prevalence (≈ 80%) [48].

Within the Bundibugyo District bat fly population evaluated in this study, 81% of bat flies were infected with a ledantevirus. For the KYAV isolates, overall mean p-distance for even the most constrained gene (*N*) was 5.6 ± 0.41%. The greatest average distance was in the *P* gene (8.4 ± 0.5%), whereas *M*, *G*, and *L* genes all had a mean p-distance of ≈ 6%. Genome-wide, average dN/dS was low (dN/dS = 0.23 ± 0.18), indicating a predominant pattern of purifying selection. However, we detected episodic diversifying selection along 6 of 35 branches of the KYAV phylogeny from this population using aBSREL (Figure 3). Viral endosymbionts of insects, such as rhabdovirid sigmaviruses, diversify within the population as host resistance alleles evolve [49]. Therefore, the diversity we have documented may have evolved as viral lineages were transmitted vertically and co-diverged with their bat fly host lineages. Unfortunately, accurate evolutionary rate estimates for ledanteviruses and their bat fly hosts do not currently exist, hindering inferences about the timing of such co-divergence events [50].

Documentation of this diversity of bat fly viruses in a single roost consisting of a man-made structure (a culvert under a road) is noteworthy. The Bundibugyo District roost was substantially larger ( > 200 bats) than the peridomestic roost approximately 80 km away in Kibale National Park, Uganda, where KYAV was originally discovered (6 bats). Therefore, a larger roost may simply support a larger diversity of viruses. Additionally, the Angolan soft-furred fruit bat is broadly distributed across equatorial Africa, and gene flow has been detected between populations as distant from each other as Côte d’Ivoire and southeastern Democratic Republic of the Congo [51]. This gene flow may, in turn, drive continent-wide mixing of African bat fly populations, as is seen in South-eastern Asia [52]. Finally, bats of other species occupy the Bundibugyo District roost, although none collected were host to ectoparasites. Therefore, co-roosting of bat species in this biogeographic “mixing zone” [53] may also enhance viral diversity.

Nycteribiid bat flies are eyeless, wingless obligate parasites of bats [1]. Their obligatory hematophagy makes them highly likely vectors for blood-borne viruses and other microorganisms [13]. For instance, these flies are known to transmit *Polychromophilus* spp. and *Nycteria* spp. plasmodiid haemospororid blood parasites [54,55], and are believed to vector *Bartonella* spp. alphaproteobacteria [56]. Bats, bat flies, and fungal hyper-parasites of bat flies have co-evolved as a tripartite system [57]. Ledanteviruses are strongly associated with bats [7], but many bat-associated ledantevirus lineages are represented by only single isolates from bats, raising doubts about their transmission ecology [11]. Group C ledanteviruses and perhaps other “bat-associated viruses” may similarly persist in coevolved tripartite systems of bat, bat fly, and virus. If so, bat-associated ledanteviruses, like some entomopathogenic rickettsiae [58], could be viewed as vertically transmitted endosymbionts of insects that at times evolve into arthropod-borne pathogens of vertebrates. That we detected KYAV in the oral swab of only one bat, despite heavy bat fly infestation across all individuals collected, re-enforces this notion. For ledanteviruses, bat flies may therefore not fit neatly into either the role of “vector” or a “reservoir,” as traditionally defined [59].

The unusual reproductive biology of bat flies may facilitate vertical transmission of microorganisms. Bat flies are adenotrophically viviparous, with a single larva developing inside the female, which feeds that larva by secretions from intrauterine milk glands [1]. Certain bacterial endosymbionts that have co-evolved with bat flies are localized in milk gland tubules, and vertical transmission of these endosymbionts is believed to be mediated through milk gland secretion [60,61]. Future studies of tissue tropism may indicate that this mechanism of vertical transmission also applies to ledanteviruses.

Overall, our results indicate that bat fly populations can harbor significant diversity of viruses, and that these viruses are likely predominantly vertically transmitted from insect to insect, but that they can occasionally infect bats and be shed orally. The presence of many KYAV variants and the divergent BUGV in the same roost at the same time, and even on the same bats, offers further evidence that bat flies may be central to a proper understanding of the cryptic ecology and evolution of the “bat-associated” viruses. Bat fly virus discovery efforts have been limited relative to those of most other hematophagous ectoparasites [13]. With further study, we may discover that other anecdotally “bat-associated” viruses are in fact associated with the ubiquitous yet poorly studied bat flies or other, even less studied, bat ectoparasites.

## Figures and Tables

**Table 1:** Distribution of bat flies and associated ledantevirus isolates on bats. KYAV: Kanyawara virus; BUGV: Bughendera virus.

Bat ID (§)	# of Flies	% Virus Pos.	Fly ID (1)	Virus ID	Fly ID (2)	Virus ID	Fly ID (3)	Virus ID	Fly ID (4)	Virus ID	Fly ID (5)	Virus ID
BF1	0	−	−	−	−	−	−	−	−	−	−	−
BF2	0	−	−	−	−	−	−	−	−	−	−	−
BF3	4	50%	BF301	KYAV−BF301	BF302	n/a	BF303	KYAV−BF303	BF304	n/a	−	−
BF4	3	100%	BF401	KYAV−BF401	BF402	BUGV	BF403	KYAV−BF403	−	−	−	−
BF5	0	−	−	−	−	−	−	−	−	−	−	−
BF6	2	100%	BF601	KYAV−BF601	BF602	KYAV−BF602	−	−	−	−	−	−
BF7	5	100%	BF701	KYAV−BF701	BF702	KYAV−BF702	BF703	KYAV−BF703	BF704	KYAV−BF704	BF705	KYAV−BF705
BF8	3	67%	BF801	KYAV−BF801	BF802	KYAV−BF802	BF803	n/a	−	−	−	−
BF9	3	67%	BF901	KYAV−BF901	BF902	KYAV−BF902	BF903	n/a	−	−	−	−
BF10	2	50%	BF1001	KYAV−BF1001	BF1002	n/a	−	−	−	−	−	−
BF11 *	4	100%	BF1101	KYAV−BF1101	BF1102	KYAV−BF1102	BF1103	KYAV−BF1103	BF1104	KYAV−BF1104	−	−

§: BF = Bundibugyo frugivore. Bat fly and virus isolate IDs are coded to indicate the fly’s bat host ID (BF(×)), and order of fly collection (BF(×)01,...02,...03). * individual with ledantevirus positive oral swab.
